# Olfactory schwannoma in an adolescent: a case report

**DOI:** 10.1007/s00247-025-06227-0

**Published:** 2025-04-05

**Authors:** Rita de Sousa, Francisco Miguel Rodrigues, Carolina Maia, Alice Carvalho, Inês Luz, Gustavo Soares, Ana Margarida Novo, Olinda Rebelo, Stephan Frank, Jürgen Hench, Sílvia Carvalho

**Affiliations:** 1 Neuroradiology, Medical Imaging Department, Unidade Local de Saúde Coimbra, Praceta Professor Mota Pinto, Coimbra, 3004-561 Portugal; 2 Pediatric Oncology Department, Pediatric Hospital, Unidade Local de Saúde Coimbra, Coimbra, Portugal; 3 Pediatric Neurosurgery Department, Pediatric Hospital, Unidade Local de Saúde Coimbra, Coimbra, Portugal; 4 Neuropathology Department, Unidade Local de Saúde Coimbra, Coimbra, Portugal; 5 Pathology Department, Universitätsspital Basel, Basel, Switzerland

**Keywords:** Adolescent, Anosmia, Anterior cranial fossa, Brain neoplasms, Ethmoid bone, Neurilemmoma, Neuroimaging, Olfactory groove

## Abstract

Olfactory schwannoma is a very rare tumor, whose origin is still under investigation. We report the case of a 16-year-old adolescent who presented with worsening headaches, hyposmia, and a convulsive crisis. Brain imaging showed a frontobasal lesion, attached to the cribriform plate, with solid and multiseptated cystic components. The patient underwent complete resection of the lesion. A comprehensive molecular study confirmed a methylation pattern consistent with schwannoma. Although rare, olfactory schwannomas should be considered in the differential diagnosis of anterior cranial fossa tumors. Given its benign nature, a complete resection leads to favorable long-term outcomes.

**Graphical Abstract:**

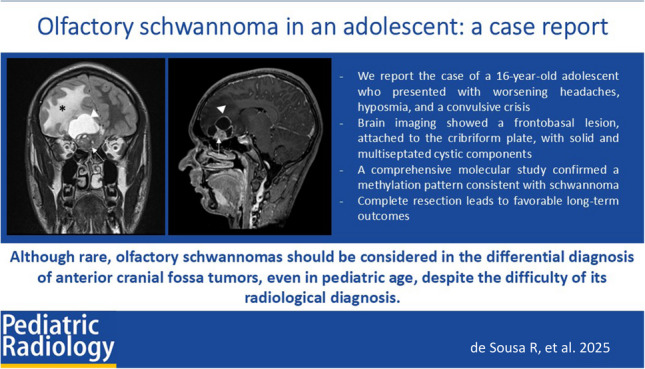

## Introduction

Schwannomas are benign tumors that arise from Schwann cells, which are present in peripheral and cranial nerves III-XII [1–3]. Intracranial schwannomas account for 8% of all intracranial tumors, with the vestibulocochlear nerve being the most commonly involved [2, 3].

Olfactory schwannomas, located in the anterior cranial fossa, are extremely rare tumors that present with radiological and clinical features similar to meningiomas [1, 4]. Their origin remains controversial as, theoretically, the olfactory nerve lacks Schwann cells [1–3, 5].

We aim to present a rare case of olfactory bulb schwannoma from our center, with olfactory dysfunction, identified by a comprehensive molecular study, and successfully treated. Informed consent was obtained from the patient for publishing the case report.

## Case report

A 16-year-old boy presented to our institution with self-limited seizures and a 3-month history of worsening holocranial headaches. The patient reported a decreased sense of smell for weeks. Neurological examination revealed no additional alterations. No relevant family medical history was known. Additionally, there were no stigmata of neurofibromatosis.

**Fig. 1:**
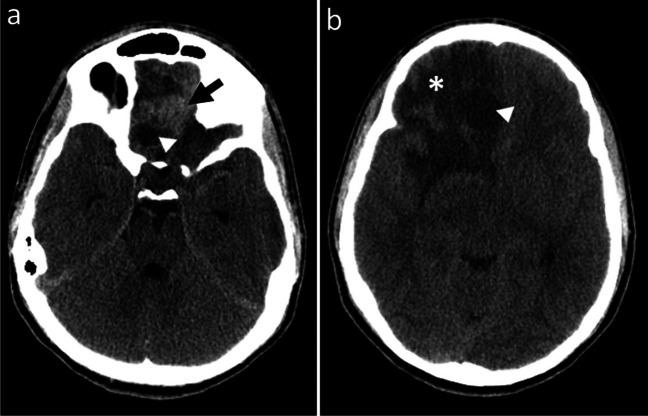
Axial non-contrast computed tomography images of the brain in a 16-year-old boy show a right frontobasal lesion. **a** Slice at the frontobasal level shows a solid hyperdense portion (*arrow*) and cystic component (arrowhead). **b** Slice at a higher level reveals the cystic component (arrowhead), accompanied by extensive vasogenic edema (asterisk) in the right frontal region

Emergency brain computed tomography (CT; SOMATOM Definition AS, Siemens Healthineers, Erlangen, Germany) revealed a heterogeneous right frontal space-occupying lesion, accompanied by extensive vasogenic edema (Fig. 1). No underlying bony alterations were identified. The patient was started on sodium valproate and dexamethasone.

**Fig. 2:**
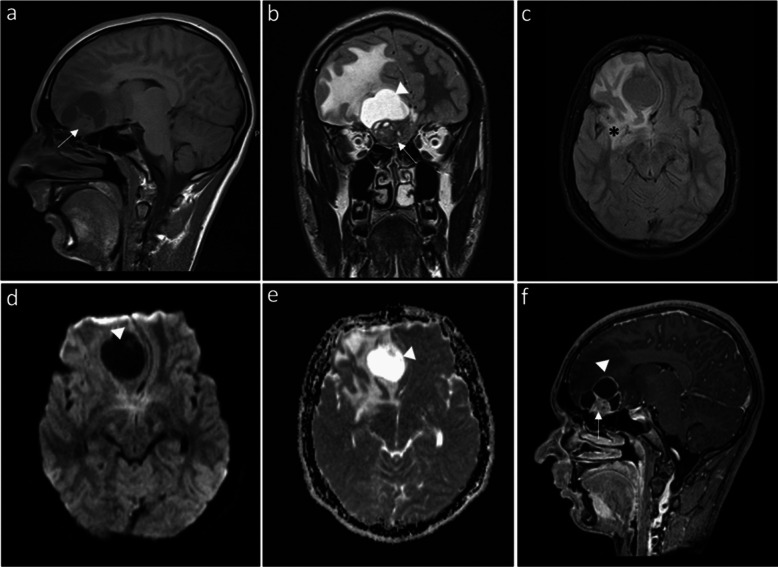
Brain magnetic resonance imaging in a 16-year-old boy shows a right extra-axial frontobasal tumor, attached to the cribriform plate, with a solid inferior component and a superior multiseptated cystic component. **a** Sagittal T1-weighted image shows the solid portion (*arrow*), isointense to the cortex. **b** Coronal T2-weighted image reveals a heterogeneously hypointense solid portion (*arrow*) with hyperintense microcystic foci and the superior cystic component (arrowhead). **c** T2-FLAIR image shows marked right frontal vasogenic edema (asterisk). **d**, **e** Axial diffusion-weighted image and corresponding apparent diffusion coefficient map demonstrate facilitated diffusion in the cystic component (arrowheads). **f** Sagittal post-contrast image shows marked enhancement of the solid portion (*arrow*) and peripheral linear enhancement of the cystic component (arrowhead)

Magnetic resonance imaging (MRI; Magnetom Espree 1.5 T, Siemens Healthineers, Erlangen, Germany) (Fig. 2) confirmed the presence of an extra-axial frontobasal tumor, attached to the cribriform plate, with a solid inferior component and a superior multiseptated cystic component. The solid component was isointense to the cortex on T1-weighted images (T1-WI) and heterogeneously hypointense on T2-weighted images (T2-WI), with hyperintense microcystic foci. The solid component of the tumor exhibited intense, slightly heterogeneous enhancement. Marked right frontal vasogenic edema was noted and there was strong mass effect. An olfactory groove meningioma was considered.

The patient was hospitalized for further monitoring and investigation. Surgical intervention was considered the best therapeutic approach.

During surgery, the tumor showed to have a purplish infiltrative intraparenchymal component with cystic cavities as well as an extra-axial, encapsulated, fibrotic, and highly vascularized component, strongly attached to the cribriform plate. The patient underwent complete tumor resection via craniotomy with a transcortical approach. There were no procedure-related complications.

The patient was discharged with his olfaction restored, no recurrence of seizures, and no further complaints.

The histopathological study revealed a tumor composed of sheets and lobules of large, round cells showing immunopositivity for SOX-10 and S100, and a Ki- 67 index of 5–6%. A comprehensive molecular study was performed. The methylation profile was compatible with a schwannoma. Given the relatively low proliferative rate and the circumscribed nature of the tumor, a watchful-waiting strategy was adopted.

**Fig. 3:**
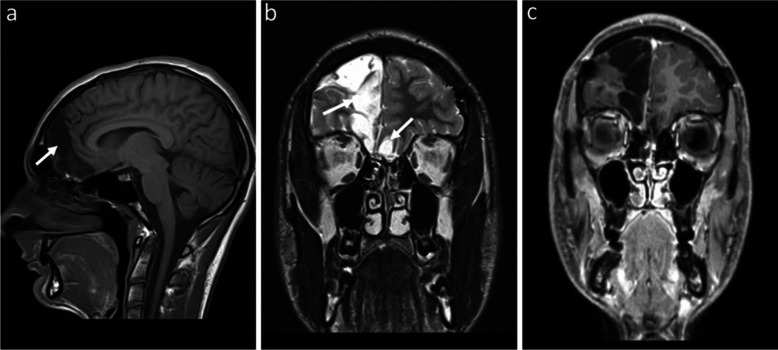
Follow–up brain magnetic resonance imaging in an 18-year-old boy, 2 years after complete tumor resection. **a** Sagittal T1-weighted image shows right frontal and frontobasal encephalomalacia (*arrow*). **b** Coronal T2-weighted image shows right frontal and bilateral frontobasal encephalomalacia (*arrows*) as sequelae of prior surgery. **c** Coronal gadolinium-enhanced T1-weighted image shows no abnormal enhancement. No radiological recurrence was detected

After 2 years, the patient is doing well, with no complaints and no signs of radiological recurrence (Fig. 3).

## Discussion

Olfactory, subfrontal, or olfactory groove schwannoma is a very rare entity, with only six pediatric cases reported worldwide, whose origin is not yet fully understood [1, 5]. Two main theories have been proposed to explain its development. The developmental theory suggests that mesenchymal pial cells transform into ectodermal Schwann cells or that neural crest cells might migrate to these regions. The non-developmental hypotheses defend that these schwannomas arise from Schwann cells present in adjacent structures, such as the anterior ethmoidal nerve, nerve plexus, or meningeal branches of the trigeminal nerve. Another possibility is that Schwann cells could arise from multipotent mesenchymal cells following injury [2, 5].

The most common manifestations of olfactory schwannomas include headache, anosmia, and seizures, all of which were present in our patient [1, 2, 5]. As they are located in the anterior fossa in relation with the olfactory system, olfactory dysfunction is a common clinical manifestation. Nevertheless, there are reported cases of preserved olfactory function [3]. Headache and seizures result from increased intracranial pressure.

The average age of onset for olfactory schwannomas is 33 years old, which contrasts with the age of our patient. These tumors are more frequent in males [1].

Schwannomas present as an iso- or hypodense mass on CT. On MRI, they can be iso- or hypointense on T1WI and heterogeneously hyperintense on T2WI, often with prominent heterogeneous contrast enhancement, particularly in larger tumors due to cystic areas [4, 6].

Preoperatively, olfactory schwannomas are frequently misdiagnosed as olfactory groove meningiomas, olfactory neuroblastomas, or dural-based metastases, the most frequent differential diagnosis for extra-axial anterior fossa neoplasms [1, 2, 6].

Some bone erosion or remodeling, and multiple low signal foci on T2*, related to microbleeds, seem to be more indicative of schwannomas rather than meningiomas, which typically show bone sclerosis and a dural tail [4, 6]. These differentiating features were not present in our case. Aggressive bone destruction and invasion of adjacent structures are warning features suggesting sinonasal malignant neoplasms.

The distinction between olfactory schwannomas and olfactory ensheathing cell tumors remains challenging, as they appear in the same topography and exhibit the same radiological features. Further molecular markers are needed for better discrimination. The latter is even rarer, as the olfactory ensheathing cells, the glial cells that cover the axons of the olfactory nerve, appear to be resistant to neoplastic transformation [6, 7].

Given the benign nature of olfactory schwannomas, the treatment of choice is complete surgical resection of the tumor, with no need for adjunctive therapy. After complete resection, the prognosis tends to be favorable [3, 7].

Although rare, olfactory schwannomas should be considered in the differential diagnosis of anterior cranial fossa tumors, even in pediatric age, despite the difficulty of its radiological diagnosis.

## Data Availability

No datasets were generated or analysed during the current study.
